# Health Care Spending: Changes in the Perceptions of the Australian Public

**DOI:** 10.1371/journal.pone.0157312

**Published:** 2016-06-13

**Authors:** Jane Robertson, David A. Newby, Emily J. Walkom

**Affiliations:** Department of Clinical Pharmacology, School of Medicine and Public Health, The University of Newcastle, Callaghan, NSW, Australia; National Institute of Health, ITALY

## Abstract

**Background:**

Increasing demand for services and rising health care costs create pressures within the Australian health care system and result in higher health insurance premiums and out-of-pocket costs for consumers.

**Objective:**

To measure changes in consumer views on the quality of the Australian health care system, contributors to rising costs and attitudes towards managing these costs.

**Methods:**

Two computer-assisted telephone interviews were conducted in 2006 (533 respondents) and 2015 (1318 respondents) and results compared.

**Results:**

More respondents in 2015 rated the Australian health care system ‘*very adequate’* than in 2006 (22.3% vs 8.3%; Odds Ratio OR 3.2, 99% CI 2.1, 5.1) with fewer ‘*concerned*’ or ‘*fairly concerned*’ about the health care costs (69.0% vs 85.7%; OR 0.37, 99% CI 0.25, 0.53). The 2015 respondents were more likely to identify new treatments for cancer (77% vs 65.7%; OR 1.75, 99% CI 1.30, 2.35) and community expectations for access to the latest technologies (73.8% vs 67%; OR 1.39, 99% CI 1.04, 1.86) as contributors to rising health care costs. While more 2015 respondents agreed that patients should pay a greater part of the health care costs, this remained a minority view (37.9% vs 31.7%; OR 1.32, 99% CI 0.99, 1.76). They were less likely to agree that doctors should offer medical treatments regardless of the cost and chance of benefit (63.6% vs 82.9%; OR 0.36, 99% CI 0.25, 0.50).

**Conclusions:**

Satisfaction with the Australian health care system has increased over time. Consumers recognise the cost pressures and have lower expectations that all services should be provided regardless of their costs and potential benefit. Public consultation on the allocation of health care resources and involvement in health care decision-making remains important. There should be community consultation about the principles and values that should guide resource allocation decisions.

## Introduction

Ageing populations, longer life expectancy, and increased rates of chronic diseases and cancers are creating increasing demand for health care services and contributing to rising health care costs worldwide. In Australia, governments struggle to meet demands for access to new and expensive medicines, health technologies, surgical interventions and hospital care, with regular claims that the increases in health care costs are unsustainable [1,2]. Views differ on the extent to which the cost increases might be attributed to consumer behaviours and inefficiencies in the health care system, changes in Australian government policies over time, or expansion of services coverage to include assessment and care planning items to better manage chronic diseases [1,3–6]. Regardless of the cause, these pressures on the Australian health care system manifest as longer hospital waiting lists, higher patient co-payments, rising private health insurance premiums and problems for patients in meeting out-of-pocket health-related expenses [7,8].

Medicare provides access to medical and hospital services for all Australian residents [9]. The program provides free or subsidized treatment by health professionals including general medical practitioners, specialists and optometrists and provides free treatment for public patients in a public hospital. Patients variably contribute to the cost of medical and hospital services through additional co-payments depending on their socio-economic circumstances, the willingness of their health care providers to accept scheduled fees as the basis of their billing, and their use of private health care services. Consumers can take supplementary private health insurance to help manage some of these additional costs. Access to pharmaceuticals is via the taxpayer funded Pharmaceutical Benefits Scheme (PBS) [10]. Patients pay a fixed amount or co-payment for each prescription, the amount depending on their social welfare status. A ‘safety net’ cuts in with heavily discounted prescriptions after a certain number have been dispensed in a calendar year. Of the estimated AU$140.2 billion health expenditure in Australia in 2011–2012 (representing around 9.5% of gross domestic product [GDP]), almost 70% was funded by governments, with 17% paid by patients and 8% by private health insurers [6].

Repeated public polls on taxation and social service provision in the 1990s and early 2000s showed the high and increasing importance of health to the Australian public and support for increasing expenditure on health [11]. In parallel, many Australians believed the standard of health care services had declined over time, although it is unclear the extent to which this might reflect more critical views of health standards or reactions to regular negative media attention on the state of public hospitals and Medicare [11]. With complex funding and health delivery models, health policy is frequently part of the national debate. This has led to calls to improve the health system, greater efforts to promote clinically and cost-effective health care and reduce wasteful expenditure, better use of the health workforce, review of provider payment models and greater emphasis on preventive health [12–15].

In 2006, we conducted a computer-assisted telephone interview (CATI) to measure consumer views on the quality of the Australian health care system, contributors to rising costs and attitudes towards managing these costs [16]. We repeated the telephone survey in 2015, and in this report we compare the responses of two representative samples of Australian consumers surveyed nine years apart to identify changes in their perceptions and attitudes over time.

## Materials and Methods

Details of the 2006 survey conducted for us by the Hunter Valley Research Foundation (HVRF) are reported elsewhere [16].

The questions used in 2015 formed part of the omnibus CQUniversity (CQU) National Social Survey (NSS-2015), a large national telephone survey of the Australian general public conducted by the Population Research Laboratory, CQUniversity, Australia. The survey includes a standardized introduction, questions reflecting the specific interests of researchers and demographic and core health questions. The 2015 survey was pilot-tested on 40 randomly-selected households with subsequent modifications based on interviewer comments.

### Ethics statement

Both 2006 and 2015 surveys were approved by the University of Newcastle Human Research Ethics Committee. Informed verbal consent to participate in the study was recorded at the time of the telephone interviews. Verbal consent is a standard procedure used by the HVRF and CQU based on extensive experience conducting telephone surveys of this type; requiring written consent produces low response rates and survey samples unlikely to be representative of the study population. This consent procedure was approved by the CQU and University of Newcastle Research Ethics Committees (Approval numbers H14/09-203 and H-2015-0213 respectively).

### Participants

In 2006, households in New South Wales, Australia were randomly selected from the Electronic White Pages. Respondents were aged 18 years and over and were randomly selected based on age relative to other householders (e.g. youngest, second oldest).

Respondents for the NSS-2015 were a random selection of persons aged 18 years or older living in Australia at the time of the survey who could be contacted by direct-dialled landline or mobile telephone service. The random digit dialling databases were supplied by Sampleworx Pty Ltd (http://www.sampleworx.com.au). Mobile telephone numbers were included recognising the growing proportion of the Australian population without landline telephones. For mobile telephone numbers, the eligible respondent was the person receiving the call (if aged ≥ 18 years). In the case of landline telephone numbers, participants were chosen based on gender to ensure an equal, random selection of males and females. Respondents were selected on the most recent birthday when there was more than one male/female in the household.

### Outcome measures

In both 2006 and 2015 surveys, participants were asked to assess the quality of health care services in Australia using a 5-point scale (*very poor* to *very adequate*) and their concern about the costs of providing health care in Australia on a 4-point scale (*not at all concerned* to *concerned)*. The remaining questions on contributors to increasing health care costs and responsibilities for managing these were reported using 5-point Likert scales (*strongly disagree* to *strongly agree*). In order to ensure comparability of results we used the same wording of questions in both 2006 and 2015 surveys.

### Statistical analysis

NSS-2015 data were tabulated and cleaned using SPSS version 22. Weighting was used to adjust for survey non-response and non-coverage where particular groups were over- or under-represented in the final sample of 1318 respondents. We compared results using weighted and unweighted data. Differences were small and therefore unweighted estimates are presented here.

A companion research study in the NSS-2015 examined respondents’ experiences with hospital care, with 474 participants (36%) reporting spending one or more nights in hospital in the past 3 years. We stratified our responses based on this definition of hospitalization and compared responses for the hospitalized and not hospitalized groups in *post-hoc* exploratory analyses for key questions where we believed recent direct exposure to the health care system may have moderated views and attitudes.

Given some evidence of age-related differences in responses in the 2006 study, we conducted exploratory *post-hoc* analyses stratifying participants to those aged <60 years and ≥60 years.

Descriptive statistics were used to summarise the survey data. Responses for each item are reported separately. Differences between groups are reported as odds ratios (OR) with the 2006 responses as the reference group. Because we performed multiple comparisons we were conservative in our calculations and report here the odds ratios with their 99% confidence intervals. StatsDirect Statistical Software Version 2.8.0 (2013) was used for all calculations.

## Results

In 2006, there were 533 completed interviews of 826 telephone contacts, giving a response rate of 64.5%.

Of 4134 telephone contacts in the 2015 study, there were 1318 respondents with complete survey data giving a response rate of 31.9%. Almost 48% of the participants were contacted using mobile telephone numbers. There were 2009 refusals to participate (48.6% of contacts) with an average time to complete the NSS-2015 of 33 minutes. The estimated sampling error, assuming a 50/50 binomial percentage distribution, is ± 2.7 percentage points.

The mean age of the 2006 and 2015 respondents was comparable (52 vs 53 years). However there were more male respondents (46% vs 37.5%) and more respondents aged ≥60 years (36.8% vs 31.9%) in the 2015 survey. (Table 1) Those reporting previous hospitalization were slightly older than those not hospitalized in the last three years (55 vs 50 years).

**Table 1:** Characteristics of survey respondents 2006, 2015.

Characteristic	2006 (N = 533)	2015 (N = 1318)
Male respondents (%)	200 (37.5)	602 (46)
Contacted using mobile phone number	n/a	48%
Mean age in years	52	53
Age range in years	18–89	18–100
Aged ≥ 60 years (%)	170 (31.9)	485 (36.8)

n/a not applicable

### General views on health care services in Australia

The Australian health care system was rated ‘*very adequate’* by 294 respondents (22.3%) in 2015 compared to 44 respondents (8.3%) in 2006 (Odds Ratio OR 3.2, 99% CI 2.1, 5.1). Fewer respondents in 2015 reported being ‘*concerned*’ or ‘*fairly concerned*’ about the health care costs compared to 2006 (69.0% vs 85.7%; OR 0.37, 99% CI 0.25, 0.53).

### Views on causes of increasing health care costs

Compared to 2006, respondents in 2015 were more likely to identify new treatments for cancer (77% vs 65.7%; OR 1.75, 99% CI 1.30, 2.35) and community expectations for access to the latest technologies (73.8% vs 67%; OR 1.39, 99% CI 1.04, 1.86) as contributors to rising health care costs (Table 2). They were less likely to identify doctor’s reluctance to refuse patient requests for drugs or tests as contributors (42.1% vs 52.7%; OR 0.65 99%CI 0.50, 0.86). Almost two-thirds of respondents in both surveys agreed that external pressures from lobby groups and pharmaceutical industry promotion were contributing to increasing costs.

**Table 2:** Contributors to rising health care costs.

Survey item	n (%) agreeing or strongly agreeing with the statement		Odds ratio* (99% CI)
	2006 (N = 533)	2015 (N = 1318)	
***New advances and practice issues***			
The development of expensive new medicines	408 (76.5)	967 (73.4)	0.85 (0.61, 1.15)
New treatments for cancer	350 (65.7)	1015 (77.0)	1.75 (1.30, 2.35)
***Social context***			
Community expectations for access to the latest technologies	357 (67.0)	973 (73.8)	1.39 (1.04, 1.86)
Doctors’ reluctance to refuse patient requests for tests and drugs	281 (52.7)	555 (42.1)	0.65 (0.50, 0.86)
Patients expecting a test or prescription at every doctor’s visit	343 (64.4)	861 (65.3)	1.04 (0.79, 1.38)
***External pressures***			
Lobby group and patient pressure to fund particular diseases	320 (60.0)	845 (64.1)	1.19 (0.90, 1.57)
Drug company advertising to persuade people to ask for medicines (in newspapers, television)	351 (65.9)	795 (60.3)	0.79 (0.59, 1.04)
Drug company promotion to doctors to prescribe medicines	354 (66.4)	824 (62.5)	0.84 (0.63, 1.12)

*2006 responses are the control group; CI confidence interval

### Attitudes towards health care costs

The 2015 respondents were more likely to agree that patients should pay a greater part of the health care bill although the difference was of borderline statistical significance (37.9% vs 31.7%; OR 1.32, 99% CI 0.99, 1.76, Table 3). More agreed that it was *not* the doctor’s responsibility to be concerned about the costs of health care to society (43.2% vs 34.7%; OR 1.43, 99% CI 1.08, 1.90).

**Table 3:** Attitudes towards health care costs.

Survey item	n (%) agreeing or strongly agreeing with the statement		Odds ratio* (99% CI)
	2006 (N = 533)	2015 (N = 1318)	
Patients should pay a greater part of the health care bill so they will be more cost-conscious	169 (31.7)	500 (37.9)	1.32 (0.99, 1.76)
The *Government* should educate the public about the true costs of health care	495 (92.9)	1179 (89.5)	0.65 (0.38, 1.07)
*Doctors* should educate their patients about the true costs of health care	395 (74.1)	880 (66.8)	0.70 (0.52, 0.95)
Only the treating physician and the patient should decide if a treatment ‘is worth the cost’	360 (67.5)	911 (69.1)	1.08 (0.81, 1.43)
No matter how small the chance of benefit, the physician should offer a medical treatment regardless of the cost	442 (82.9)	838 (63.6)	0.36 (0.25, 0.50)
It is *not* the doctor’s responsibility to be concerned about the costs of health care to society	185 (34.7)	570 (43.2)	1.43 (1.08, 1.90)
The public should be consulted about rationing decisions and allocation of health care resources	375 (70.4)	955 (72.5)	1.11 (0.82, 1.49)

*2006 responses are the control group; CI confidence interval

Notably, 2015 respondents were less likely to agree that doctors should offer a medical treatment regardless of the cost and chance of benefit (63.6% vs 82.9%; OR 0.36, 99% CI 0.25, 0.50).

### Hospitalized versus non-hospitalized respondents

More hospitalized than non-hospitalized respondents rated the health care system as ‘*very adequate’*, were concerned about the costs of providing health care services, identified costs of cancer treatments as a contributor to health care costs, reported that patients should pay a greater part of the health care bill and responded that only the treating physician and patient should decide if the treatment is worth the cost (Table 4). However these differences were mostly small and were not statistically significant.

**Table 4:** Consumer responses with and without recent hospitalization.

Survey item	n (%) agreeing or strongly agreeing with the statement		Odds ratio* (99% CI)
	Hospitalized# (N = 474)	Not hospitalized (N = 844)	
Australian health system ‘*very adequate’*	122 (25.7)	172 (20.4)	1.35 (0.94, 1.93)
Concern about costs (*concerned or fairly concerned*)	337 (71.1)	572 (67.8)	1.17 (0.84, 1.63)
New treatments for cancers	372 (78.5)	643 (76.2)	1.14 (0.79, 1.65)
Patients should pay a greater part of the health care bill	184 (38.8)	316 (37.4)	1.06 (0.78, 1.45)
Only the treating physician and the patient should decide if the treatment is worth the cost	342 (72.2)	569 (67.4)	1.25 (0.90, 1.75)
No matter how small the chance of benefit the physician should offer a medical treatment regardless of the cost	302 (63.7)	536 (63.5)	1.00 (0.74, 1.38)

* No hospitalization responses are the control group; CI confidence interval

^#^ spending one or more nights in hospital in the past 3 years

### Responses stratified by age

Compared to younger respondents, those aged ≥60 years were statistically significantly more likely to assess the health care system as *very adequate*, recognise the cost impacts of new treatments for cancer, suggest that patients may a greater portion of the health care bill, and agree that only the treating physician and patient should decide if treatment was worth the cost (Table 5). The older group were less likely to consider that the physician should offer a treatment regardless of cost and how small the chance of benefit.

**Table 5:** Consumer responses by age group (<60 years; ≥60 years).

Question	n (%) agreeing or strongly agreeing with the statement		Odds Ratio* (99% CI)
	Age <60 years (N = 821)	Age ≥60 years (N = 485)	
Australian health system ‘*very adequate’*	147 (17.9)	146 (30.1)	1.97 (1.38, 2.82)
Concern about costs (*concerned or fairly concerned*)	564 (68.7)	335 (69.1)	1.02 (0.74, 1.41)
New treatments for cancers	613 (74.7)	394 (81.2)	1.47 (1.01, 2.15)
Patients should pay a greater part of the health care bill	279 (34.0)	215 (44.3)	1.55 (1.14, 2.11)
Only the treating physician and the patient should decide if the treatment is worth the cost	534 (65.0)	368 (75.9)	1.69 (1.21, 2.38)
No matter how small the chance of benefit the physician should offer a medical treatment regardless of the cost	548 (66.7)	284 (58.6)	0.70 (0.52, 0.96)

* age <60 years is reference category, age data were missing for 12 respondents; CI confidence interval

## Discussion

The greater satisfaction with health care quality and lower concern about the costs of providing health care in 2015 compared to 2006 was unexpected. However the NSS-2015 findings are consistent with the significantly improved views of the Australian health care system reported in successive Menzies–Nous Australian Health Surveys conducted in 2008, 2010 and 2012 [17] and a national Newspoll survey conducted in 2011 [18]. In the most recent of the Menzies-Nous surveys, over 85% of Australians expressed confidence that they would receive quality and safe medical care, effective medication and the best medical technology if they were seriously ill. It is possible that the attention given to good governance and accountability in the health sector in the past decade, along with the establishment of national safety and quality health service standards and accreditation of health care facilities, have provided both greater awareness of minimum standards and reassurance to the general public of the quality of health care available to Australian citizens [19,20]. The Newspoll survey (a representative sample of 1207 of the Australian public) found that 89% of respondents were somewhat or very confident they would receive high quality and safe medical care on becoming seriously ill, reflecting the high standards of the Australian health care system. However, respondents expressed concerns about health care costs, with 21% lacking confidence they would be able to afford care [18].

This confidence that the system will deliver high quality care is notwithstanding repeated media reporting that the health system is “*on* [the] *way to 'catastrophe'* “[21]), “*on* [the] *critical list”* [22], at “*breaking point”* [23], and that “*billions* [are] *`wasted' in health system”* [24]. Costs are “a *bitter pill*: *medical bills rise for all”* [25] and “*a fiscal risk”* [26]. There are warnings that “*the oldies are on their way*, *so is our care sustainable*?*”* [27]. Sceptical consumers may be ignoring the repeated claims of politicians and other spokespeople with vested interests, and tuning out from negative media reports, believing that governments will find the money required given the importance of health and the recurring demands for higher government spending and better services [11, 28]]. Others have argued that Government claims of unsustainability are unfounded and reflect an underlying belief that user-pay health systems are better [29]. These authors have challenged the assumptions that market forces increase efficiency, increase quality, and that price signals work to moderate demand in health care.

The emphasis of consumer complaints has shifted over time to uncoordinated or fragmented care across health care sectors that contribute to waste, inefficiencies, and perceived ineffectiveness of health care services [28]. The rapidly growing demands of an ageing population have also focused attention on nursing homes and residential aged-care facilities where service provision has not matched needs or satisfied expectations regarding quality of care [17]. The scale of the challenges facing the Australian health care system is reflected in the numbers of reviews examining system frailties [30]. These reviews cover mental health, primary health care, reforms of funding of services to deal with the demands of chronic disease and changes in medical treatment and technology, national pharmacy agreements, private health insurance and administrative efficiencies to deal with rising costs and fragmented systems [30].

High costs for new medicines for the treatment of a range of cancers including melanoma and treatment for hepatitis C amongst others are regularly reported in the media, and have been the subject of Government Committee special enquiries [31,32]. Concerns about costs and value of some new cancer treatments mirror concerns internationally [31,33–38]. The possibilities of long-term disease control and cure with many of the new cancer therapies have generally not been realized, prompting calls for priority-setting and evidence-informed decision-making in this highly emotive area of health care [38,39]. A US Institute of Medicine workshop noted that “as cancer care evolves, we need a system that is rational and not rationed”[40].

Consumers have an important role in debates about service provision across the cancer care continuum and the difficult balance between affordable access to medicines of sometimes marginal clinical benefit and denying patients the hope that treatment might offer. Perhaps consumers are recognizing the challenges, with fewer respondents in our 2015 survey agreeing that doctors should offer a medical treatment regardless of the cost and chance of benefit. In parallel with more public discussions of the limits of health care, there must be improvements in support services for end-of-life care [41].

Consumer behaviours are also influential with a substantial majority of respondents in both surveys agreeing that patient expectations for access to new technologies, for tests and prescriptions were contributing to increasing costs. Patients’ requests and expectations, and prescribers’ perceptions of these, are strong influences on prescribing behaviour [42]. Suggested strategies for moderating these expectations include clarifying the patient’s concerns and treatment goals, discussing management options, and evidence-informed shared decision-making [42]. However these are time consuming tasks and with the pressures of short appointment times, acceding to patient requests may be quicker and easier.

While more respondents in our 2015 survey and more older respondents agreed that patients should pay a greater part of the health care bill to encourage more cost-conscious behaviours, this remains a minority view. The evidence on the impact of co-payments is mixed, with some arguing that price signals are ill-fitted to the health care system [28,43]. Australian Government attempts to introduce an AU$7 co-payment for general practitioner visits in 2014 were met with widespread criticism, with the proposals for the “widely loathed fee” withdrawn in 2015 [44–46].

Both surveys identified high levels of support for greater awareness of the true costs of health care and greater consultation with the general public about rationing and resource allocation decisions. These are not matters for health care professionals alone; however the challenges lie in finding meaningful ways to engage the public in the difficult dialogue on the value, costs and prioritization of health services. Citizen juries involving “disinterested members of the public (citizens) … [rather] than experienced ‘service-users’ (patients or consumers)” is one method proposed of public engagement, although relatively little is known about how consumer perspectives influence the deliberations and recommendations of these juries [47]. There is limited evidence of the impact of public involvement in health-care policy development and the costs and benefits of such engagement, although such involvement may be seen to have intrinsic value [48,49]. Moving from implicit to explicit consideration of rationing will require clear articulation of the ethical principles and procedures for the fair allocation of limited resources [50].

### Study limitations

The lower response rate in 2015 (31.9% vs 64.5%) may relate in part to consumer fatigue with unsolicited telephone calls often for marketing purposes, with 2009 refusals from 4134 telephone contacts (48.6%). However steep rates of decline in participation rates for telephone surveys have been reported elsewhere [51,52]. It is unclear the extent to which non-response has introduced bias in responses. It was not possible to collect information on non-respondents; however, consistency with responses in 2006 and small differences in weighted and unweighted estimates in the 2015 survey suggest that non-response biases are not a significant concern in this study.

The 2015 questionnaire took slightly longer (average 33 minutes) as it was part of an omnibus national social survey (NSS-2015) and included several question sets from different researchers along with introductory text, screening questions and collection of demographic data. In contrast, the 2006 survey was a stand-alone study. However, the questions reported here were asked in the first half of the NSS-2015 and therefore we do not believe that respondent fatigue and loss of interest in the survey would have adversely affected responses despite the overall longer length of the 2015 survey.

There were differences in the gender distribution of respondents in the two surveys as a result of differences in selection procedures. In 2006, respondents were selected on the basis of relative age in the household and this resulted in more women than male respondents. It may also reflect greater willingness of women to participate in such surveys, a feature reported in other telephone interview studies [53]. In 2015, participants were selected on gender, resulting in an equal random selection of males and females. However, there was no evidence that men and women differed in their views on the topics covered in the survey.

## Conclusions

The importance of good health care will ensure that health policy and funding for the health care system will remain topics for national debate in Australia. While there have been improvements in levels of consumer satisfaction with the Australian health care system over time, only a minority of the public regard the health care system as ‘*very adequate*’. Consumers increasingly recognise the cost pressures on the health system and have lower expectations that all services should be provided regardless of their costs and potential benefits. Consumers believe that the public should be consulted about rationing decisions and allocation of health care resources and express willingness to be involved in health care decision-making. The challenge is to find meaningful ways to engage the general public in the discussions and decision-making around resource allocation. At a minimum, there should be community consultation about the principles and values that should guide resource allocation decisions.

## Supporting Information

S1 Dataset Survey response data from 2006 and 2015, Excel format. (XLSX)
